# Synthetic Implant Migration Generation for Accuracy and Precision Evaluation of AI-Based CT-RSA in Total Hip Arthroplasty

**DOI:** 10.3390/diagnostics16101484

**Published:** 2026-05-14

**Authors:** Hassan M. Nemati, Albin Christensson, Andreas Pettersson, Gunnar Flivik

**Affiliations:** 1 Ortoma AB, 412 85 Gothenburg, Sweden; andreas.pettersson@ortoma.com; 2 Department of Orthopedics, Skåne University Hospital, Clinical Sciences, Lund University, 221 84 Lund, Sweden; albin.christensson@med.lu.se (A.C.); gunnar.flivik@med.lu.se (G.F.)

**Keywords:** medical image processing, Ortoma treatment solution, computer assisted, synthetic implant migration, AI-based CT-RSA, hip implant migration

## Abstract

**Background/Objectives**: Radiostereometric analysis (RSA) is the gold standard for measuring implant migration, with CT-RSA increasingly used as an alternative. To evaluate CT-RSA, it is important to assess data that include the surrounding soft tissues, rather than data from simplified phantoms, while also avoiding unnecessary radiation from multiple scans. This study proposes a method for generating multiple follow-up CTs from a single post-operative CT (baseline CT) by simulating stem migration and uses it to assess an AI-based CT-RSA tool. **Methods**: The method involves extracting the stem implant voxels from the baseline CT, digitally translating them along the *x*-, *y*-, and *z*-axes, and storing the result as new follow-up CTs. The voxel spacing of the baseline CT is used to define the ground-truth translations, which are then compared with the AI-based CT-RSA results using descriptive statistics and Bland–Altman plots. **Results**: Using 10 patients’ baseline CTs, 780 follow-up CTs were generated. Bland–Altman analysis showed a mean difference of 0.00 mm, largest LoA −0.10 to 0.09 mm, and translational precision for zero-migration of 0.026 to 0.049 mm. **Conclusions**: The proposed method offers a practical alternative to phantom-based models, and the AI-based CT-RSA showed high accuracy and precision for stem translation. The study addresses translational migration only.

## 1. Introduction

Radiostereometric analysis (RSA) is the gold standard for measuring implant migration because of its high accuracy and precision [1,2]. RSA provides three-dimensional measurements with sub-millimeter accuracy and is used to monitor early implant migration and predict long-term implant stability [3].

However, RSA has significant practical limitations. RSA requires tantalum markers inserted in the bone, a calibration cage, dedicated biplanar X-ray equipment, trained personnel, and specialized analysis software [4]. Due to these requirements, RSA is available only at a limited number of specialized centers, which restricts routine clinical use.

CT-based implant migration measurement, also known as CT-RSA [5], is a more accessible and lower-cost alternative compared to the specialized equipment required for RSA [6,7,8,9,10,11,12]. Validating CT-RSA is, however, challenging, and current approaches each have limitations. Comparison with RSA provides clinically relevant ground truth but is constrained by the need for specialized equipment, tantalum marker implantation, and the inherent precision limits of RSA itself [4]. Double examination protocols, in which patients are scanned twice with repositioning, allow precision estimation but yield no true migration signal and introduce additional radiation exposure [6,11,12]. Phantom-based studies offer known displacements and have been used to benchmark CT-RSA methods [8], but phantoms cannot fully reproduce the soft tissues, patient-specific bone morphology and metal-implant artifacts found in clinical CT images. Furthermore, constructing and scanning physical phantoms is resource-intensive and does not scale easily across different implant types or scanner configurations. A patient-based validation framework is therefore needed, one that provides ground-truth migration data without extra radiation and keeps the anatomical complexity of clinical CT.

Synthetic data generation is one way to address the limited data available in medical imaging research. These methods are typically based on generative models such as generative adversarial networks, variational autoencoders and diffusion models that aim to produce realistic synthetic images. A recent review by Koetzier et al. [13] reviews how synthetic medical images are used for data augmentation, generalization of trained models, and simulation of rare cases. They also raise reliability concerns that generative models may not preserve the co-appearance of local and global pathological features.

Our method does not use machine-learning-based image generation. Instead, implant migration is simulated directly by shifting the CT voxels of the stem implant along the *x*-, *y*-, or *z*-axis while keeping the rest of the anatomy unchanged. Because the rest of the image is untouched, the original HU distribution and anatomical context of the scan are preserved; only the position of the implant changes. With this method, a small number of post-operative CT scans can be used to generate hundreds of follow-up CTs with known, controlled stem migration, and without exposing patients to additional radiation.

The synthetic migration datasets were used to evaluate the accuracy and precision of an AI-based CT-RSA tool (OTS™ Hip Follow-up, Ortoma AB, Gothenburg, Sweden). The tool uses AI-based segmentation together with a model-based implant-alignment step to detect bones and implants automatically, so migration is measured without manual input. In earlier work it was shown that the non-model-based version performs comparably to RSA [14], and that the model-based version (referred to here as AI-based CT-RSA) gives results close to those of model-based RSA [15]. In the current study, the performance of this tool was assessed using synthetically generated migration data, with migration measurements compared against ground-truth displacement values derived directly from the applied voxel translations.

The study addresses translational migration only.

## 2. Materials and Methods

The methodology for generating synthetic migration of a stem implant (synthetic migration generation) contains several steps which are described in detail in the following. To capture variability in patient characteristics, scanner settings, and implant size and subtype, 10 baseline CT scans from patients who had undergone THA were used.

For each of the baseline CTs, synthetic migration was generated, and the resulting images were stored as follow-up CTs. Subsequently, both the baseline CT and the follow-up CTs were processed using the AI-based CT-RSA. The AI-based CT-RSA operates independently of the synthetic migration generation and has no prior knowledge of the applied implant displacement.

### 2.1. Patient Data

The data used in this study include 10 different patients (mean age 61.1 years, 5 females) with primary osteoarthritis of the hip who underwent total hip replacement from November 2020 to November 2021 with an uncemented Pinnacle cup and Corail stems (Pinnacle^®^, Corail^®^, DePuy Synthes, Warsaw, IN, USA). The baseline CTs are low-dose scans of the pelvis taken within two days after surgery (following mobilization). The scanning protocol varied, and two different brands of CT-scanners were used, resulting in differences in the administered dose. However, the radiation exposure was kept well below that of a standard pelvis CT [16].

Table 1 summarizes patients’ characteristics and Table 2 summarizes the scanner characteristics along with the type and size of the used implant.

### 2.2. Study Design

#### 2.2.1. Synthetic Migration Generation

**Figure 1:**
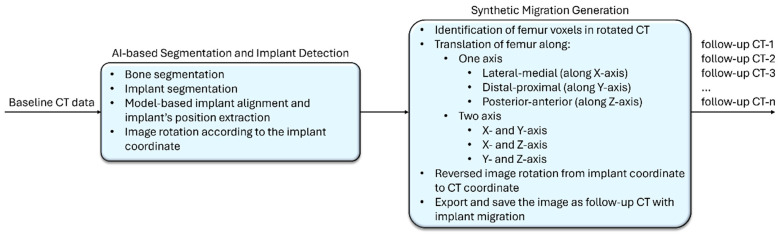
Workflow of synthetic migration generation, generating follow-up CTs with synthetic migration.

The workflow diagram for synthetic migration generation is illustrated in Figure 1. At first, each baseline CT was imported into the AI-based segmentation and implant detection algorithms (the algorithms are part of OTS Hip Plan module [17]). Here, the bones were automatically detected and labeled as FemurLeft, FemurRight, HipLeft, and HipRight. The treatment-side stem implant was then automatically segmented and labeled as StemLeft or StemRight, depending on the operated side.

Based on the recorded implant information from the patient’s surgery (stem size and type), the CAD model of the stem was selected and aligned to the segmented model using a model-based alignment algorithm. This process calculated the transformation matrix that aligned the CAD model of the stem to the segmented stem in the patient.

**Figure 2:**
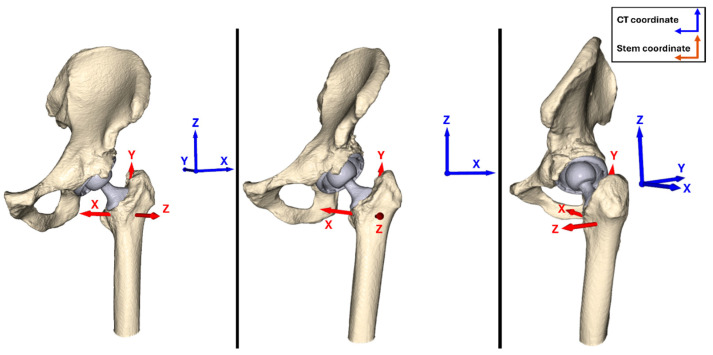
An example of the output of AI-based segmentation and implant detection applied to a baseline CT with an implant in the left side is presented. The red arrows indicate the stem coordinate system, while the blue arrows represent the CT coordinate system.

Next, key landmarks from the CAD model, such as the stem center, tip, and neck position, were extracted. These landmarks were then used to establish the stem coordinate system, where the longitudinal axis of the prosthetic stem (centerline) was defined as the *y*-axis, and the neckline was defined as the *x*-axis. An illustration of the difference between CT coordinate system and stem coordinate system is presented in Figure 2. To enhance readability, subscript ‘ct’ (i.e., $x_{ct},y_{ct},\;and\;z_{ct}$) is used to denote the CT coordinate system, while coordinates without a subscript (*x*, *y*, and *z*) refer to the stem coordinate system.

**Figure 3:**
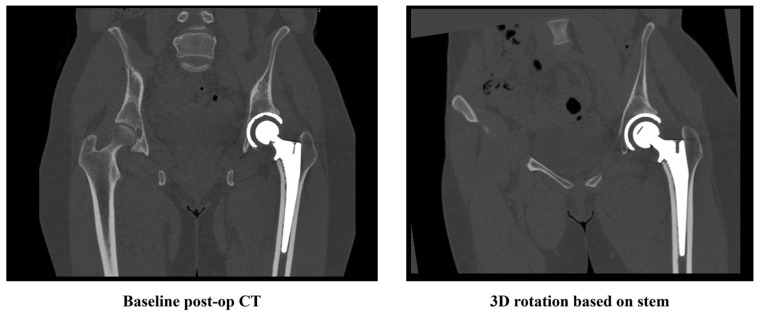
Screenshot of a CT slice image before and after applying 3D rotation based on the stem coordinate.

To ensure that the simulated migrations in synthetic follow-ups are along the stem axis, the baseline CT was rotated in 3D to align with the stem’s orientation. This adjustment made the *y*-axis of the rotated CT image parallel to the femoral stem and the *x*-axis parallel to the neckline. Figure 3 presents a screenshot of a slice image from a sample CT scan, displayed both before and after rotation.

Considering the CT image as a 3D voxel array of Hounsfield Unit (HU) values, the migration of the stem implant was simulated in a controlled manner by shifting the voxels corresponding to the segmented stem along the *x*-, *y*-, or *z*-axis, while keeping the voxels corresponding to the other body parts intact. The original stem voxels that were no longer part of the stem after the shift were filled using the smoothing window function, assigning the nearest neighbor soft-tissue HU.

To generate synthetic migration, all voxels in the rotated CT image associated with the stem implant (stem and head) were selected and shifted, first along one axis, then along two axes at a time.

Stem migration along one axis in stem coordinate system includes:

- Nine migration steps along *x*-axis (lateral-medial), with steps of 1 voxel,
  dx={−4,−3,−2,−1,0,1,2,3,4}.
- Eleven migration steps along *y*-axis (distal-proximal), with steps of 1 voxel,
  dy={−5,−4,−3,−2,−1,0,1,2,3,4,5}.
- Nine migration steps along *z*-axis (posterior–anterior), with steps of 1 voxel,
  dz={−4,−3,−2,−1,0,1,2,3,4}.

The zero-voxel shift corresponds to the absence of stem shift, i.e., zero migration.

**Figure 4:**
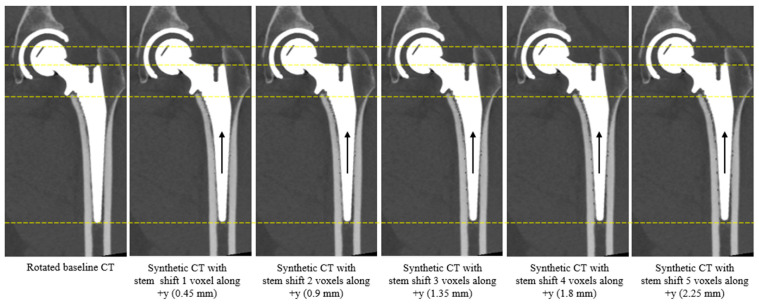
Zoomed-in screenshot of CT slice image from rotated baseline CT and rotated follow-up CTs with synthetic migration of stem along *y*-axis with 1 voxel increment. For this baseline CT, the voxel spacing along the *y*-axis is 0.45 mm. The dashed lines are added to aid visualization of the stem movement. The black arrow shows the direction of stem implant movement.

The maximum shift was 5 voxels along the *y*-axis, compared with 4 voxels along the other two axes, because of the smaller voxel spacing in that direction. The spacing along the *y*-axis (corresponding to $z_{ct}$ in CT coordinate system) was smaller compared to the other two axes (see Table 2). Among all the 10 baseline CTs, the maximum displacement along *x*- and *z*-axis (corresponding to $x_{ct}$ and $y_{ct}$ in CT coordinate system) was 3.44 mm (4 voxels, 4 × 0.8594 = 3.44), while the maximum displacement along the *y*-axis (corresponding to $z_{ct}$ in CT coordinate system) was 3 mm (5 voxels, 5 × 0.6 = 3). The range of stem migrations was chosen to represent potential implant migration magnitudes [18]. An example of the shift along positive *y*-axis is illustrated in Figure 4.

Stem migration along two axes includes:

- Sixteen migration steps in both *x*- and *y*-axes, from +4 voxels to −4 voxels, excluding the zero-voxel shift.
- Sixteen migration steps in both *x*- and *z*-axes, from +4 voxels to −4 voxels, excluding the zero-voxel shift.
- Sixteen migration steps in both *y*- and *z*-axes, from +4 voxels to −4 voxels, excluding the zero-voxel shift.
- One example with no stem translation, zero-voxel shift.

For the translation in two axes, the amount of voxel movement at each iteration along the two-axes $\left(a,b\right)$ is defined as

(1) $$d_{ab}=\{(a,b)|a\in \left\{-4,-3,-2,-1,1,2,3,4\right\},b\in \left\{a,-a\right\}\}$$

Accordingly, from one baseline CT, 29 different synthetic translations were generated from the movement in one axis, and 49 different synthetic translations from the movement in two axes (in total 78 synthetic follow-up CTs). The amount of voxel translation with information about the voxel spacing from the baseline CT was considered as the ground-truth migration.

Finally, each of the follow-up CTs with the synthetic stem movement was rotated back to the CT coordinate using the inverse transformation matrix of the stem (rotated back from *x*, *y*, and *z* stem coordinate to $x_{ct},y_{ct},\;and\;z_{ct}$ CT coordinate) and saved as a new CT image and named as follow-up CT-1, follow-up CT-2, …, follow-up CT-*n*.

All these steps were performed in Python 3.9.

#### 2.2.2. Automatic Migration Measurement Using AI-Based CT-RSA

**Figure 5:**
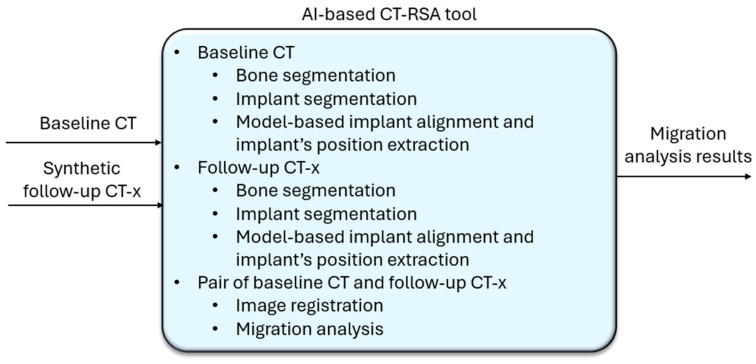
Workflow of AI-based CT-RSA.

Each follow-up CT-x along with the baseline CT was sent to the AI-based CT-RSA tool (see Figure 5). The AI-based CT-RSA first performed AI-based segmentation and implant detection on both the follow-up CT-x and the baseline CT. Next, a model-based implant alignment algorithm was applied to each image to align the CAD model to the segmented model and extract key landmarks.

Following this, an additional image alignment algorithm was performed to align the segmented femur in the follow-up CT-x with the segmented femur in the baseline CT (femur-to-femur alignment). The transformation results from this alignment mapped the treatment-side femur in the follow-up CT-x to the baseline CT. By comparing the stem position after the femur-to-femur alignment, the migration of the stem implant was measured.

Translational movements are presented in millimeters and rotations in degrees. Migrations are analyzed along the three axes in the stem coordinate system of the baseline CT. The results are presented as if all hips were right-sided. Thus, positive directions for translations are medial, superior, and anterior translation along the *x*-, *y*-, and *z*-axes respectively. Positive directions for rotations are anterior tilt (*x*-axis), internal rotation (*y*-axis), and valgus (*z*-axis).

The AI-based CT-RSA tool does not use tantalum markers. It segments bones and implants automatically with built-in machine-learning models and extracts anatomical landmarks that drive a model-based implant alignment. The segmentation models are based on Convolutional Neural Networks (CNNs), developed and validated on a large dataset of hip patient CT data comprising more than 140,000 images. The models were trained and tested on data covering a range of patient anatomies, implant types, and scanner configurations to support generalization. On-the-fly data augmentation (random rotations, flips, intensity shifts, and Gaussian noise) was applied during training to further improve robustness against scanner variability and metal-artifact distortions. The underlying CNNs achieve a mean Dice score above 0.97 for bone labels. Because the software is proprietary, architectural details (layer structure, loss functions, and optimizer) are not disclosed; however, the tool’s performance has been independently validated in earlier clinical studies [14,15].

**Figure 6:**
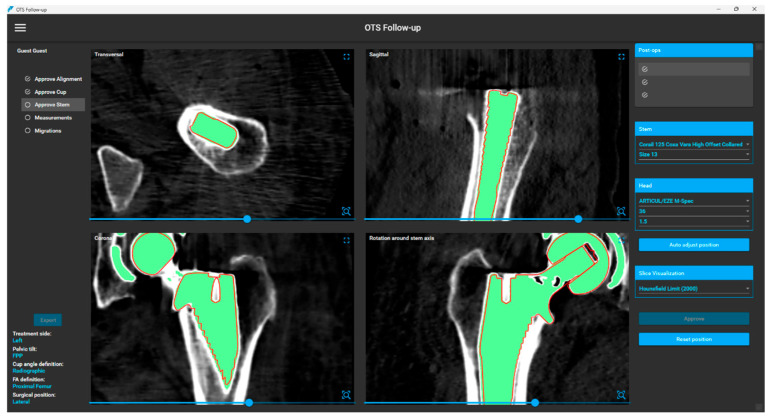
Overview of the AI-based CT-RSA tool for stem implant detection. The segmentation of the implant is visually represented in green, while the contour of the implant’s CAD model, following subsequent alignment, is depicted in red.

AI-based CT-RSA performs migration measurement automatically in less than 5 min (per pair of follow-up CT-x and baseline CT) on a standard computer equipped with a dedicated NVIDIA graphic processing unit (GPU) with 4 GB memory and NVIDIA GPU computing capability greater or equal to version 7. Figure 6 presents an example of the results obtained from the stem segmentation and the subsequent alignment with the stem CAD model in 3D.

#### 2.2.3. Evaluation Metric and Statistics

The accuracy is evaluated by calculating the difference between the ground-truth migration values derived from the voxel spacing information and the measurement results obtained from the AI-based CT-RSA. For synthetic migration along specific axes, it is expected that the results generated by the AI-based CT-RSA correspond closely to the ground-truth along those axes, and for the axes where voxel movement is zero, the expected outcome is zero migration. Thus, the accuracy of the tool was assessed by comparing the non-zero migration values, while the precision was evaluated based on the zero migrations with results represented as 1.96 times the standard deviation (SD), for the 95% confidence interval.

Normality of the difference between AI-based CT-RSA and the ground-truth was assessed with the Q-Q plot.

The agreement between the AI-based CT-RSA and the ground-truth data was evaluated using Bland–Altman plots [19]. In the Bland–Altman plots, the amount of migration is represented on the *x*-axis and the signed difference between the ground-truth and the AI-based CT-RSA on the *y*-axis. This makes any systematic bias in the AI-based CT-RSA easy to see. Limits of agreement (LoAs) were calculated as mean difference ± 1.96 × SD.

Statistical analyses were performed in Python.

## 3. Results

In total, 780 follow-up CTs with synthetic migration were generated; 90 follow-up CTs with stem translation along lateral-medial direction (*x*-axis); 110 follow-up CTs with stem translation along distal-proximal direction (*y*-axis); 90 follow-up CTs with stem movement along posterior-anterior direction (*z*-axis); and 490 follow-up CTs with stem translation along two axes at the same time.

### 3.1. Migration Along One Axis

**Figure 7:**
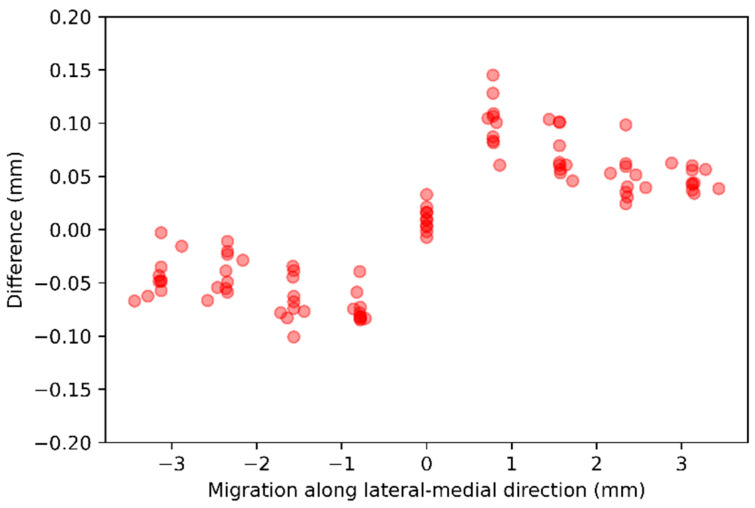
The scatter plot representation of the difference between the ground-truth and the AI-based CT-RSA while the stem voxels were shifted 1 voxel along the lateral-medial direction.

Migration along *x*-axis—For each of the 10 baseline CTs, nine follow-up CTs were generated, with stem voxels shifted along the lateral-medial direction in one-voxel steps from −4 to +4 voxels. The difference between the ground-truth and the AI-based CT-RSA is summarized in Table 3 and illustrated as scatter plots in Figure 7.

**Figure 8:**
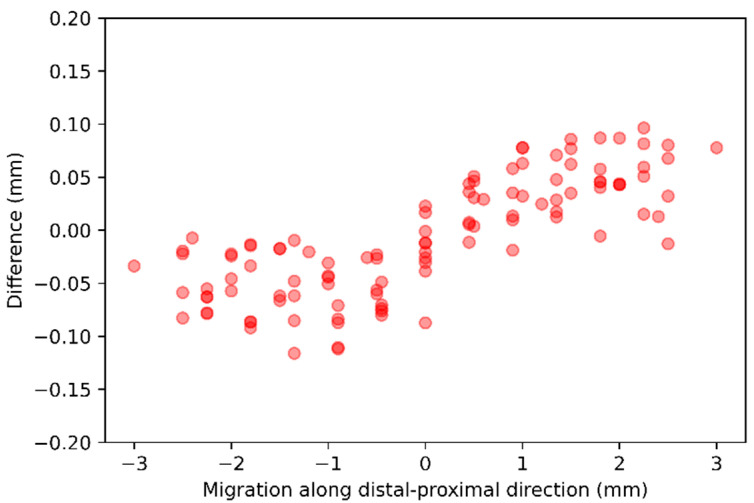
The scatter plot representation of the difference between the ground-truth and the AI-based CT-RSA while the stem voxels were shifted 1 voxel along the distal-proximal direction.

Migration along *y*-axis—For each of the 10 baseline CTs, 11 follow-up CTs were generated, with stem voxels shifted along the distal-proximal direction in one-voxel steps from −5 to +5 voxels. The difference between the ground-truth and the AI-based CT-RSA is summarized in Table 4 and illustrated as scatter plots in Figure 8.

**Figure 9:**
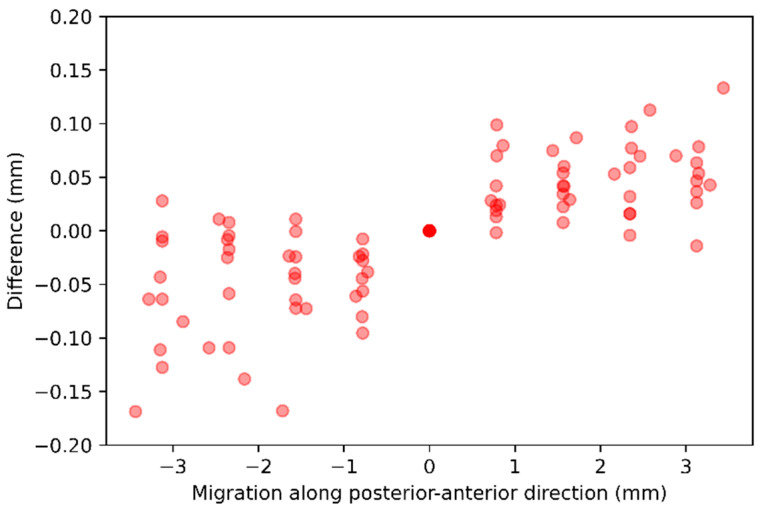
The scatter plot representation of the difference between the ground-truth and the AI-based CT-RSA while the stem voxels were shifted 1 voxel along the posterior-anterior direction.

Migration along *z*-axis—For each of the 10 baseline CTs, nine follow-up CTs were generated, with stem voxels shifted along the posterior-anterior direction in one-voxel steps from −4 to +4 voxels. The difference between the ground-truth and the AI-based CT-RSA is summarized in Table 5 and illustrated as scatter plots in Figure 9.

### 3.2. Migration Along Two Axes

For each of the 10 baseline CTs, 49 follow-up CTs were generated with synthetic migration while the stem voxels were shifted along two axes at the same time. The difference between the ground-truth and the AI-based CT-RSA is summarized in Table 6.

### 3.3. Accuracy Evaluation

**Figure 10:**
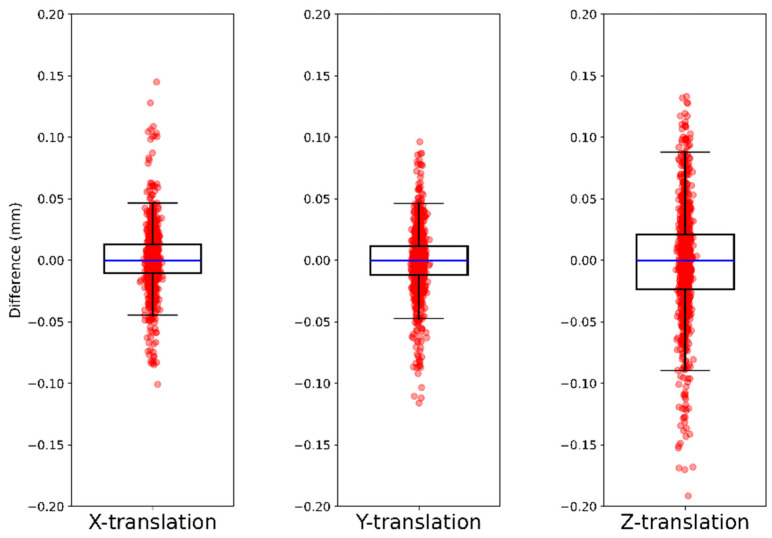
Box plot representation of the translation difference between the ground-truth and the AI-based CT-RSA. The circles represent the difference for each follow-up CT; the box indicates the first, second (median), and third quartiles where the spread is indicated with the vertical lines.

Combining synthetic migration along one-axis and two-axes results in 780 follow-up CTs. This combined migration data were compared with the ground truth and summarized in Table 7 and illustrated as box plots in Figure 10. The maximum deviation was observed in the posterior-anterior direction shift with the absolute difference of 0.191 mm.

**Figure 11:**
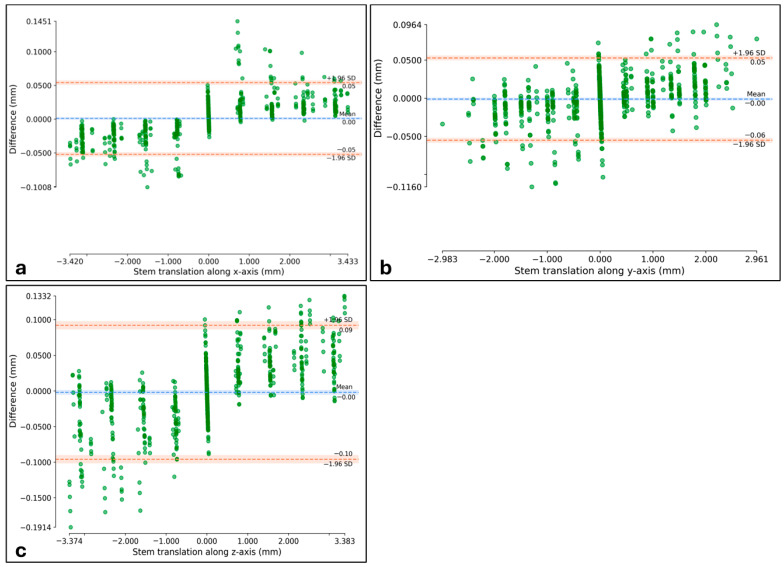
Bland–Altman plots for stem *x* (**a**), *y* (**b**), and *z* (**c**) translations. Limits of agreement are shown as dotted orange lines with 95% confidence intervals. The mean difference (bias) is shown as a dotted blue line with 95% confidence intervals. The results are calculated as the difference between the ground-truth migration values and the AI-based CT-RSA results.

For the combined data, the Bland–Altman plots showed that the mean difference between the ground-truth and the AI-based CT-RSA was 0.00 mm in all the three axes. The LoA in *x*-axis was −0.05 and 0.05 mm (Figure 11a), in *y*-axis −0.06 and 0.05 mm (Figure 11b), and in *z*-axis −0.1 and 0.09 mm (Figure 11c).

### 3.4. Precision Evaluation in Axes with Zero Migration

Precision is calculated for migrations along each axis when there is no migration on that specific axis (see Table 8). In total there were 290 follow-up CTs with zero migration: 90 follow-up CTs from synthetic migration generation along *x*-axis (zero migration along *y*- and *z*-axes), 110 follow-up CTs from synthetic migration generation along *y*-axis (zero migration along *x*- and *z*-axes), and 90 follow-up CTs from synthetic migration generation along *z*-axis (zero migration along *x*- and *y*-axes).

**Figure 12:**
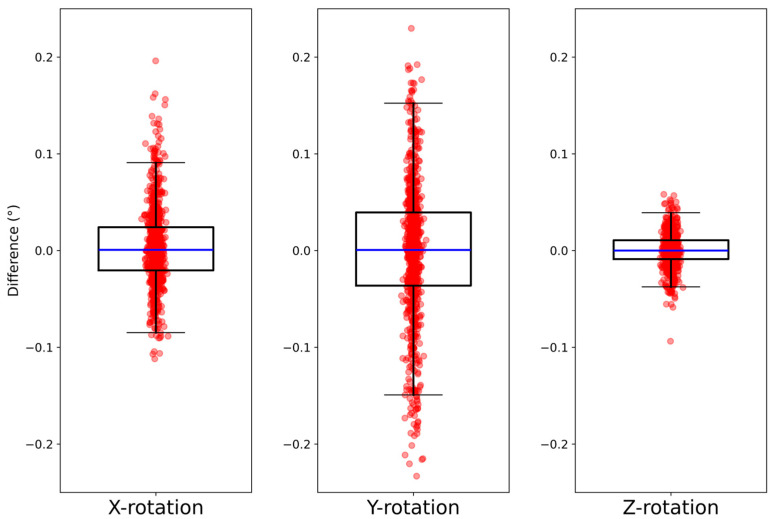
Box plot representation of the rotation difference between the ground-truth and the AI-based CT-RSA. The circles represent the difference for each follow-up CTs; the box indicates the first, second (median), and third quartiles where the spread is indicated with the vertical lines.

Since the AI-based CT-RSA also evaluates rotational changes, the precision in measuring zero rotation is also reported. A summary of the findings of all 780 follow-up CTs is provided in Table 9 and illustrated in Figure 12. The precision of the AI-based CT-RSA in detecting rotational differences is lower than that for translational differences.

## 4. Discussion

To our knowledge, this is the first method that generates controlled implant migration synthetically from post-operative CT scans while keeping the full anatomy in the field of view, including soft tissues and their HU values. A limited set of post-operative CT scans was used to generate hundreds of follow-up CTs. The approach is applicable to post-operative CT scans across implant types and scanner models. The resulting synthetic datasets were used to evaluate the accuracy and precision of an AI-based CT-RSA for stem migration.

In a previous study [15], the accuracy of the AI-based CT-RSA was assessed in detecting stem and cup migration by comparing its results to those obtained from RSA. Ten patients who underwent THA surgery were scanned post-operatively and analyzed using the AI-based CT-RSA and model-based RSA at postoperative intervals of 3, 12, and 24 months. The precision of the AI-based CT-RSA was also evaluated through double examinations on 14 patients, involving two scans per patient with repositioning conducted between the scans. That study indicated that the AI-based CT-RSA is a viable alternative to RSA, with better precision, less operator dependence, and wider accessibility. In the current study, the performance of the AI-based CT-RSA was assessed with synthetic implant migration data.

While RSA is considered the gold standard for measuring implant migration, it is both expensive and time-consuming, requiring dedicated equipment and an expert operator. As a result, only a limited number of hospitals can perform RSA. In the recent literature, CT-RSA is increasingly used as an alternative to RSA, with broadly comparable precision. Table 10 provides a summary of key studies and advancements in this field.

Some studies focus on marker-based CT-RSA, while others explore marker-free CT-RSA that relies solely on bone surface anatomy. Marker-free CT-RSA has shown better precision than marker-based methods for most translations and rotations [6,10,11,12,20,21]. Three studies developed a phantom device to establish controlled displacement as the ground truth for evaluation [8,20,22], while others based their analysis on patient data [6,10,11,12,14,15,21]. Additionally, some studies compared CT-RSA results directly with RSA outcomes [8,12,14,15]. In a review by Kärrholm [23], RSA precision within the 99% significance interval in clinical studies was reported to range from 0.15 to 0.60 mm for translations and from 0.3° to 2° for rotations. Recent CT-RSA studies have generally reported lower bias and higher precision than RSA for both translation and rotation.

**Table 10:** Summary of recent CT-RSA studies on hip implant.

Method/Description	Type	Precision CT-RSA (Range)	Precision RSA (Range)	Accuracy CT-RSA
3D CT 2016 [22]-MB precision 12 phantom comparisons accuracy 12 phantom comparisons	Cup	Tr: 0.01–0.09 mm Ro: 0.06–0.29°	Tr: 0.04–0.09 mm Ro: 0.08–0.32°	Compared to controlled displacement: Tr: 0.07–0.32 mm Ro: 0.21–0.82°
CTSA 2016 [20]-MF precision 8 phantom comparisons and 5 patients accuracy 39 phantom comparisons	Stem	Phantom: Tr: less than 0.09 mm Ro: less than 0.14° Patients: Tr: less than 0.40 mm Ro: less than 0.55°	-	Compared to controlled displacement: upper LoA Tr: less than 0.28 mm upper LoA Ro: less than 0.20°
CTMA 2020 [10]-MF precision 9 patients	Stem	Tr: 0.1–0.3 mm Ro: 0.1–0.4°	-	-
CTMA 2020 [6]–MB and MF precision 24 patients	Cup	With markers: Tr: 0.08–0.20 mm Ro: 0.20–0.43° Without markers: Tr: 0.07–0.31 mm Ro: 0.20–0.39°	-	-
CTMA 2021 [11]–MB and MF precision 10 patients	Cup	With markers: Tr: 0.10–0.16 mm Ro: 0.31–0.37° Without markers: Tr: 0.10–0.16 mm Ro: 0.21–0.31°	-	-
CTMA 2022 [12]-MF precision 20 patients accuracy 30 patients—post-op vs. 1y follow-up	Cup	Tr: 0.06–0.15 mm Ro: 0.21–0.63°	Tr: 0.06–0.13 mm Ro: 0.23–0.35°	Compared to RSA: lower LoA *y*-axis Tr: –0.22 mm upper LoA *y*-axis Tr: 0.25 mm Approximation from plotted results: lower LoA Ro: –0.65 mm upper LoA Ro: 0.52 mm
CTSA 2023 [8]-MB precision 9 phantom comparisons accuracy 31 phantom comparisons	Cup and Head	Head: Tr: 0.11–0.28 mm Ro: 0.34–0.42° Cup: Tr: 0.08–0.11 mm Ro: 0.19–0.42°	-	Compared to controlled displacement: Head Tr: 0.04–0.18 mm Ro: 0.28–0.46° Cup Tr: 0.04–0.08 mm Ro: 0.17–0.43°
Non-model-based OTS™ follow-up 2024 [14]-MF accuracy 30 patients—post-op vs. 5y follow-up	Cup and Stem	-	Cup Tr: 0.06–0.22 mm Ro: 0.4–1.2° Stem Tr: 0.18–0.4 mm Ro: 0.12–1.04°	Compared to RSA: Cup lower LoA *y*-axis Tr: –0.15 mm upper LoA *y*-axis Tr: 0.22 mm Stem lower LoA *y*-axis Tr: –0.32 mm upper LoA *y*-axis Tr: 0.67 mm
V3MA * [21] 2024-MF precision 48 patients	Cup	Tr: 0.05–0.13 mm Ro: 0.06–0.22°	Tr: 0.11–0.27 mm Ro: 0.15–0.67°	-
Model-based OTS™ follow-up 2025 [15]-MF accuracy 10 patients—post-op vs. 3, 12, 24 months follow-up precision CT-RSA 14 patients precision RSA 12 for stem and 10 for cup from 9 patients	Cup and Stem	Cup Tr: 0.06–0.09 mm Ro: 0.34–0.51° Stem Tr: 0.07–0.16 mm Ro: 0.08–0.36°	Cup Tr: 0.17–0.38 mm Ro: 0.41–0.66° Stem Tr: 0.22–0.30 mm Ro: 0.24–0.63°	Compared to RSA: Cup lower LoA *y*-axis Tr: –0.126 mm upper LoA *y*-axis Tr: 0.388 mm Stem lower LoA *y*-axis Tr: –0.245 mm upper LoA *y*-axis Tr: 0.404 mm
Current study—AI-based CT-RSA (model-based OTS™ follow-up)-MF 10 patients—780 synthetic migrations	Stem	Tr: 0.03–0.05 mm Ro: 0.03–0.14°		Compared to controlled displacement: Tr: −0.19–0.13 mm

MF: marker-free; MB: marker-based; Tr: translation (mm); Ro: rotation (°); * volumetric matching micromotion analysis.

In the phantom study by Scheerlinck et al. [20], the precision of CT-RSA for assessing stem implant migration was evaluated using a stem implant placed in a dry macerated human femur. The reported translational and rotational precision for zero migration was less than 0.09 mm and less than 0.14 degree, respectively. These values provide a benchmark for the precision of CT-RSA in controlled conditions. In the current study, the translational and rotational precision of our method for zero migration was 0.05 mm and 0.14 degree, respectively, demonstrating comparable precision relative to the phantom-based study by Scheerlinck et al. [20].

Furthermore, when comparing the results from the proposed methodology with data from our previous study on real patients [15], which both used the same model-based, AI-based segmentation and implant detection algorithms, the current study shows improved precision. Similarly, the observed differences in precision are consistent with the pattern reported by Scheerlinck et al. [20] wherein phantom-based data—free from real-world factors such as patient anatomy variability, scanner types, and other confounding influences—shows higher precision compared to patient data. These results indicate that the proposed approach not only matches existing benchmarks but also offers a scalable alternative to phantom-based studies for assessing implant migration.

A key limitation is that the resolution of the simulated movement is set by the voxel spacing of the baseline CT: shifts smaller than one voxel cannot be simulated directly. Thus, while this method effectively simulates implant migration, the voxel size inherently limits the granularity of movement steps, potentially affecting the simulation of smaller migrations.

CT voxel spacing also directly impacts the accuracy of other CT-RSA tools. Smaller voxel sizes result in higher precision imaging, which is critical for detecting subtle implant migrations. For example, in baseline CT with voxel dimensions of 0.82 × 0.82 × 0.6 mm, implant migration can be detected in increments of 0.82 mm along the *x*- and *y*-axes, and in increments of 0.6 mm along the *z*-axis. Smaller voxels generally improve precision for small implant movements, but at the cost of larger data and higher radiation dose. For example, research has demonstrated that higher-resolution voxel size (smaller voxels) yields more accurate reconstructions and measurements of implants and bone structures in dental imaging [24]. Nonetheless, this improvement comes with the trade-off of higher radiation doses and greater processing requirements.

The accuracy of the AI-based CT-RSA was assessed using Bland–Altman plots as illustrated in Figure 11. These plots do not indicate any overall bias in the evaluation. However, when examining positive and negative migration separately, a slight bias related to the direction of movement becomes apparent. Specifically, the AI-based CT-RSA shows a tendency to underestimate the actual migration. For instance, in cases where the stem translation along the *z*-axis was negative (i.e., the ground-truth translation was less than 0) across 200 follow-up CTs, the mean difference was −0.046 mm. Conversely, for cases with positive stem translation along the *z*-axis (200 follow-up CTs), the mean difference was 0.047 mm. Similar patterns were observed along other axes, although the bias was smaller in magnitude. The bias is small and unlikely to affect clinical use.

Pixel-level segmentation errors do not propagate one-to-one into the migration measurement. The segmentation labels are used as input to a model-based CAD-alignment step, which fits the implant CAD model to the segmented surface; the alignment is therefore robust to small voxel-level segmentation imperfections, since the rigid CAD model averages out local boundary noise. The femur-to-femur registration step then operates on the bone surface, which is large and rich in features compared with possible local segmentation errors. The results reported here (sub-0.05 mm precision for zero migration) are consistent with segmentation errors being well damped by the model-based alignment.

**Figure 13:**
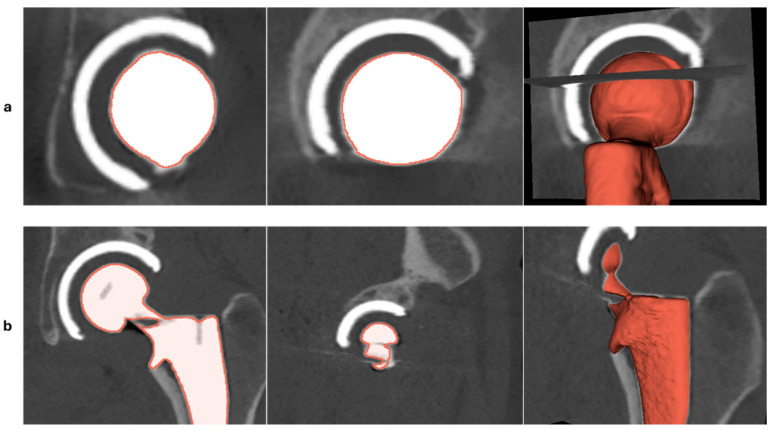
Two different baseline CTs from two different patients, one has artifacts on the head implant (**a**), and the other has artifacts on the neck of the stem implant (**b**).

The displacement errors from synthetic migrations indicated that the largest discrepancy occurred in stem translation along the *z*-axis, with a maximum absolute difference of 0.191 mm. A more detailed examination of the scans from patients with larger discrepancies showed that the baseline CT images contained artifacts caused by the metal implant components. In general, in CT imaging, metallic implants can introduce significant artifacts due to the high density and atomic number of the metal. These artifacts result in issues such as beam hardening, photon starvation, and scattering effects, which manifest as streaks, noise, and distortion around the implant, ultimately affecting image quality [25]. Screenshots of two baseline CT slices and a 3D model of the stem implant are shown in Figure 13. In CT images like the one in Figure 13a, the artifacts cause distortion around the stem head, which impacts the model-based implant alignment (CAD alignment) process and leads to a larger difference in stem translation along the *z*-axis. In contrast, the CT image shown in Figure 13b exhibits artifacts around the implant neck, which tend to have less impact on CAD alignment and, therefore, a smaller effect on the AI-based CT-RSA results. The machine-learning models behind the AI-based CT-RSA were trained on clinical CT data with varying levels of metal artifact, which gives the tool some inherent robustness to these distortions, as reflected in the accuracy and precision reported here. Nevertheless, the recommended scanning protocol for this software explicitly advises the use of metal artifact reduction filters for post-operative CT scans. Following this protocol should further reduce artifact-related errors and improve performance compared with the unfiltered conditions used here.

The zero-rotation results show that the precision of the AI-based CT-RSA for rotation is lower than for translation. However, the rotational precision remains better than that reported for RSA [1,2,3], which supports its clinical use.

The proposed synthetic-migration method also has limitations. It does not reproduce scanner variability, noise, or other real-world imaging effects. Clinical CT scanners differ between vendors in resolution, noise profile, and contrast [26]. The proposed methodology for generating synthetic migration does not directly account for these variations when creating follow-up images. Furthermore, the 780 synthetic follow-up CTs generated in this study were derived from only 10 baseline patient scans and should therefore not be interpreted as fully independent clinical samples. The diversity of the dataset is inherently limited by the number of unique patients, and results may not fully reflect the variability encountered across broader clinical populations or scanner configurations.

This study focused on stem translation. Real implant migration, however, also involves rotational changes, subsidence, tilting, bone-implant interface remodeling, and biological responses such as osseointegration or bone resorption. While the AI-based CT-RSA tool also measures rotational migration, and rotational precision results are reported here, the synthetic migration generation methodology introduced in this study was limited to translational displacements. Extending the methodology to incorporate controlled rotational perturbations of the implant voxels would be a valuable direction for future work. Similar analyses can be conducted for cup implant translation.

## 5. Conclusions

This study presents a method for generating synthetic implant migration data from post-operative CT scans, enabling controlled evaluation of CT-RSA tools without exposing patients to additional radiation. Using this approach, 780 follow-up CT images with known ground-truth displacements were generated from 10 baseline patient scans and used to assess the accuracy and precision of an AI-based CT-RSA tool. Under these controlled synthetic conditions, the AI-based CT-RSA showed accuracy and precision values within the range reported by previous phantom-based CT-RSA studies. These findings are based on synthetic validation and must be interpreted in that context. Studies with real clinical follow-up data in larger and more diverse cohorts are needed to confirm the clinical validity and generalizability of both the method and the AI-based CT-RSA tool.

## Figures and Tables

**Table 1:** Patients’ characteristics.

Feature	Value
Age (years)	
mean ± SD *	61.1 ± 8.5
Gender	
Male	5
Female	5
Treatment side	
Left	5
Right	5
BMI	
Range	20.7–31.1
Mean	24.99

* standard deviation.

**Table 2:** Scanner characteristics and stem implant type/size.

Feature	Implant Type/Size *	Scanner Manufacturer	Voxel Spacing $(x_{ct}$, $y_{ct}$, $z_{ct}$) mm **	Slice Thickness	Exposure mAs	Pitch	KV
Patient 1	KLA13	Siemens	(0.8594, 0.8594, 0.5)	0.6	25	0.8	120
Patient 2	K12A	Siemens	(0.7871, 0.7871, 0.5)	0.6	27	0.8	120
Patient 3	K12A	Siemens	(0.8203, 0.8203, 0.6)	0.6	19	0.8	120
Patient 4	K11A	Siemens	(0.7207, 0.7207, 0.5)	0.6	41	0.8	120
Patient 5	KLA10	Siemens	(0.7871, 0.7871, 0.5)	0.6	18	0.8	120
Patient 6	KLA10	Philips	(0.7812, 0.7812, 0.45)	0.9	129	0.703	120
Patient 7	K11A	Philips	(0.7812, 0.7812, 0.45)	0.9	120	0.735	120
Patient 8	K9A	Philips	(0.7812, 0.7812, 0.45)	0.9	175	0.6	120
Patient 9	KLA13	Philips	(0.7812, 0.7812, 0.45)	0.9	166	0.796	120
Patient 10	KHO13	Philips	(0.7812, 0.7812, 0.45)	0.9	87	0.703	120

* the implant name follows this naming convention: KLA13 refers to High offset 125 degrees angle (lateralised) with size 13, K12A refers to Std offset 135 degrees angle with size 12, and KHO13 refers to High offset 135 degrees angle with size 13. ** the voxel spacing information for each baseline CT is used to create controlled translation in mm along different axis.

**Table 3:** Results from synthetic migration along lateral-medial direction.

Value	Stem Translation	Mean	SD	Min	Max
90 follow-up CTs with synthetic migration	along *x*-axis	0.006	0.064	−0.101	0.145
	along *y*-axis	−0.003	0.032	−0.103	0.072
	along *z*-axis	−0.008	0.026	−0.055	0.068

**Table 4:** Results from synthetic migration along distal-proximal direction.

Value	Stem Translation	Mean	SD	Min	Max
110 follow-up CTs with synthetic migration	along *x*-axis	0.011	0.015	−0.027	0.05
	along *y*-axis	−0.007	0.055	−0.116	0.096
	along *z*-axis	−0.006	0.025	−0.064	0.092

**Table 5:** Results from synthetic migration along posterior-anterior direction.

Value	Stem Translation	Mean	SD	Min	Max
90 follow-up CTs with synthetic migration	along *x*-axis	−0.001	0.006	−0.016	0.01
	along *y*-axis	−0.002	0.013	−0.04	0.03
	along *z*-axis	−0.002	0.061	−0.169	0.133

**Table 6:** Results from synthetic migration along two axes at the same time.

Value	Stem Translation	Mean	SD	Min	Max
490 follow-up CTs with synthetic migration	along *x*-axis	−0.002	0.019	−0.059	0.047
	along *y*-axis	0.001	0.018	−0.052	0.055
	along *z*-axis	0	0.052	−0.191	0.132

**Table 7:** Results from all synthetic migration data.

Value	Stem Translation	Mean	SD	Min	Max
780 follow-up CTs with synthetic migration	along *x*-axis	0.001	0.027	−0.101	0.145
	along *y*-axis	−0.001	0.028	−0.116	0.096
	along *z*-axis	−0.002	0.048	−0.191	0.133

**Table 8:** Results from zero migration.

Value	Stem Translation	Mean	SD	Min	Max	Precision
290 follow-up CTs with zero migration	along *x*-axis (*n* = 200)	0.005	0.013	−0.027	0.05	0.026
	along *y*-axis (*n* = 180)	−0.003	0.024	−0.103	0.072	0.047
	along *z*-axis (*n* = 200)	−0.007	0.025	−0.064	0.092	0.049

Precision is calculated as 1.96 × SD.

**Table 9:** Results from zero rotation. Positive directions for rotations were anterior tilt (*x*-axis), internal rotation (*y*-axis), and valgus (*z*-axis).

Value	Stem Rotation	Mean	SD	Min	Max	Precision
780 follow-up CTs with zero rotation	along *x*-axis	0.005	0.042	−0.112	0.196	0.082
	along *y*-axis	−0.002	0.073	−0.233	0.23	0.143
	along *z*-axis	0.001	0.017	−0.094	0.058	0.033

Precision is calculated as 1.96 × SD.

## Data Availability

Data are available on request.

## Abbreviations

The following abbreviations are used in this manuscript:

RSA	Radiostereometric Analysis
OTS™	Ortoma Treatment Solution
THA	Total Hip Arthroplasty
AI	Artificial Intelligence
ML	Machine Learning
